# Outcome Measures of Chinese Herbal Medicine for Coronary Heart Disease: An Overview of Systematic Reviews

**DOI:** 10.1155/2012/927392

**Published:** 2012-04-23

**Authors:** Jing Luo, Hao Xu

**Affiliations:** ^1^Graduate School, Beijing University of Chinese Medicine, Beijing 100029, China; ^2^Cardiovascular Diseases Center, Xiyuan Hospital, China Academy of Chinese Medical Sciences, Beijing 100091, China

## Abstract

*Objective*. The aim of this overview was to summarize the outcome measures of Chinese herbal medicine (CHM) as the treatment of coronary heart disease (CHD) based on available systematic reviews (SRs), so as to display the current situation and evaluate the potential benefits and advantages of CHM on CHD. *Methods*. An extensive search included the Cochrane Database of Systematic Reviews, MEDLINE, and 4 databases in Chinese. SRs of CHM for CHD were included. Besides evaluating and summarizing the outcome measures, we also estimated the quality of the included reviews by PRISMA (preferred reporting items for systematic reviews and meta-analyses). Data were extracted according to predefined inclusion criteria by two independent reviewers. *Results*. 46 articles were included. 20 kinds of CHM were reviewed. 7 SRs were concerned with myocardial infarction (MI), 38 SRs were related to angina pectoris. 11 SRs had primary endpoints, while others focused on secondary endpoints to evaluate CHM for CHD such as angina pectoris and electrocardiogram (ECG). One SR reported more adverse effects of CHM for CHD and of the SRs analyzed quality of life. Many CHM appeared to have significant effect on improving symptoms, ECG, biomarkers and so on. However, most SRs failed to make a definite conclusion for the effectiveness of CHM in CHD patients due specifically to the poor evidence. And according to PRISMA we found most of the trials in the SRs were of low quality. *Conclusion*. Primary endpoints were not used widely. The benefits of CHM for CHD need to be confirmed in the future with RCTs of more persuasive primary endpoints and high-quality SRs.

## 1. Introduction

Coronary heart disease (CHD) is the most common cause of death in western countries. With the infectious diseases controlled and improvement of people's living, the morbidity of CHD increases year by year in many developing countries. Acute myocardial infarction (AMI) and angina pectoris are the most important two types of CHD. Chinese herbal medicine (CHM) has a 3000-year-old history with unique theories for concepts of etiology and systems of diagnosis and treatment [1]. The interest in CHM is growing rapidly beyond China [2–5]. In recent years, some researchers have reported the effect of CHM on clinical symptoms, biomarkers and mortality in CHD patients. However, the evidence of CHM needs to be reviewed systematically and appraised critically.

High-quality systematic reviews (SRs) of randomized controlled trials (RCTs) are the sources of the best evidence [6]. Currently, there is an increasing number of SRs on studies of CHM, but few of them concluded that CHM was definitely effective for CHD due to the weak evidence. In addition to rigorous clinical design and standard reporting, the selection of outcome measures also plays an important role in drawing a more persuasive conclusion. The aim of this overview was to summarize the outcome measures of CHM as the treatment of CHD based on available SRs, so as to display the current situation and evaluate the potential benefits and advantages of CHM on CHD.

## 2. Methods

Electronic literature searches were performed to identify the maximum possible number of systematic reviews/meta-analyses of CHM for CHD. The following electronic databases were searched: (1) The Cochrane Database of Systematic Reviews (Issue 10 of 12, Oct 2011); (2) MEDLINE (2001 to 2011); (3) Chinese Biomedical Database (CBM, 2001 to 2011); (4) China National Knowledge Infrastructure (CNKI, 2001 to 2011); (5) Wanfang Databases (2001 to 2011); (6) Chinese VIP Information (VIP, 2001 to 2011). CBM, CNKI, Wanfang, and VIP were databases in Chinese. We searched databases in Chinese because CHMs were researched in china mostly. And we searched papers from 2001 to 2011 for high-quality RCTs and SRs mainly focusing in recent ten years.

**Figure 1:**
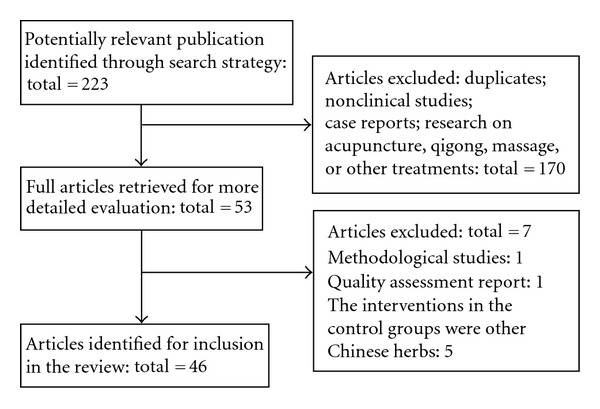
Flow-chart of SRs selection.

The strategy below was used to search The Cochrane Library and adapted appropriately for use in different electronic bibliographic databases: #1 herb*; #2 medic*; #3 (#1 and #2); #4 Chinese; #5 (#3 or #4); #6 cardiac; #7 heart; #8 circulation; #9 (#6 or #7 or #8); #10 (#5 and #9). To determine which article was we want, we scanned the title and abstract of each record independently by two reviewers (J. Luo and H. Xu). If the information included a systematic review or a meta-analysis of CHM for CHD, the full paper was obtained for further assessment. Papers were excluded when problems occurred with: repeat publication; methodological studies; quality assessment report; the interventions in the control groups were other Chinese herbs; research on acupuncture, qigong, massage, or other treatments (Figure 1).

We divided the outcome measures into primary endpoints and secondary endpoints [50, 51]. Primary endpoints include the mortality, AMI, restenosis after percutaneous coronary intervention (PCI), and recanalization. Secondary endpoints mainly indicate surrogate endpoints and laboratory measures, which include angina pectoris, arrhythmia, heart failure, consumption of nitroglycerine, electrocardiogram (ECG), ultrasonic cardiogram (UCG), Level of blood lipids, plasma endothelin, nitric oxide, myocardial enzyme, hemorheology, heart rate variability, and traditional Chinese medicine (TCM) syndrome.

In addition, we used PRISMA (preferred reporting items for systematic reviews and meta-analyses) as assessment tool to estimate the quality of the included reviews. This checklist includes 27 items of 7 key areas. And it describes the preferred way to present the abstract, introduction, methods, results, and discussion sections of a systematic review and a meta-analysis paper. It requires authors of each review to include a flow diagram that provides information about the number of studies identified, included, and excluded and the reasons for excluding them [52]. Information on each of the included reviews was imported into PRISMA statement for analysis. All data were extracted independently by two authors using predefined criteria. Disagreements were resolved by discussion between the authors. All inconsistencies were revised after a consensus was reached.

## 3. Results

46 articles were included (7 in English and 39 in Chinese). 39 SRs from the Chinese databases were published between 2004 and 2011. Since 2007, the number of SR increased markedly. 5 SRs from the Cochrane Database were published between 2006 to 2011 [8, 13, 26, 36, 44]. 2 SRs from MEDLINE were published between 2006 to 2011 [14, 45].

7 SRs were concerned with myocardial infarction (MI), 38 SRs were related to angina pectoris, and one SR was concerned with preventing and treating restenosis after PCI. The trials in SRs were mainly originated from china. The original trials included were called “RCTs” or “quasi-RCTs”, but only a few of them were typical RCTs. Most of the trials in the SRs were of low quality, only 14 RCTs were high quality: one was concerned with MI, 12 were related to angina pectoris, and one was about preventing and treating restenosis.

20 kinds of CHM were reviewed, including injections, capsules, tablets, pellets, and herbal decoction as follows: Danshen preparations (*n* = 13) [8, 14, 20–22, 33, 38–41, 45–47], 7 of them were compound salvia pellet [14, 22, 33, 38, 45–47]; Tongxinluo Capsule (*n* = 4) [13, 22, 27, 37]; Yiqi huoxue (supplementing qi and activating blood circulation) products (*n* = 3) [10, 32, 49]; Xuefu zhuyu decoction (*n* = 2) [23, 30]; herbal products (*n* = 4) [11, 12, 17, 26]; Shengmai injection (*n* = 2) [9, 24]; Suxiao jiuxin wan (*n* = 2) [35, 36]; Gingko (*n* = 2) [28, 29]; Acanthopanax (*n* = 2) [53, 54]; Puerarin (*n* = 2) [15, 44]; Shexiang baoxin wan (*n* = 2) [34, 55]; Shenmai injection (*n* = 1) [7]; Tetramethylpyrazine (*n* = 1) [43]; Shuxuetong (*n* = 1) [48]; Xinkeshu (*n* = 1) [31]; Safflower injection (*n* = 1) [25]; Rhodiola (*n* = 1) [42]; Kudiezi injection (*n* = 1) [19]; Shuyu zaogan tablets (*n* = 1) [18]; Dengzhanhua injection (*n* = 1) [16].

11 SRs analyzed primary endpoints and the others all focused on secondary endpoints to evaluate CHM for CHD (Table 1). This was mainly based on whether there were available data in the original trials or not. Four primary endpoints were analyzed in the SRs including mortality, nonfatal myocardial infarction, restenosis after PCI, and recanalization. None of these SRs analyzed the quality of life. Angina pectoris was the most common secondary endpoint in the SRs. There was one SR without clear outcome measures [53], and 2 SRs only used “marked effective,” “effective,” “ineffective” as comprehensive outcome measures involving symptoms improvement and ECG changes [19, 54]. Many CHMs appear to have significant effect on improving symptoms, ECG, and level of blood lipids and reducing the consumption of nitroglycerine, and so forth. Some SRs also reflected that CHM may be effective to reduce the risk of subsequent MI, heart failure, and arrhythmia. However, most SRs failed to draw a definite conclusion of the effectiveness of CHM for CHD due specifically to the poor evidence.

Adverse effects, which are important when evaluate a medicine, should be regarded as an essential outcome measure in clinical trials. However, only a few of the trials in the SRs had long-term data on adverse effects. Most of adverse effects of CHM were mentioned as “low adverse effect” or “none obvious”. The adverse events reported majorly were abdominal complaints, nausea, and dyspepsia. One review reported more adverse reactions in treatment groups than in control groups [44]. Recently, several reviews have highlighted adverse reactions of CHM [56, 57].

Compared the usage of outcome measures between Cochrane and non-Cochrane reviews, we found that outcome measures of the included papers in Cochrane are more comprehensive. Every Cochrane review took primary endpoints, secondary endpoints, and safety as outcome measures. However, primary endpoints and safety are seldom taken as outcome measures in most of the non-Cochrane reviews. None of reviews analyzed quality of life or pay attention to medical economics.

According to PRISMA statement, we found that most of the included reviews are of low quality. The deficiencies are as follows: review methods in the abstracts and rationales for review were not well reported; only about half of the SRs reported the characteristics of included trials; just 5 SRs provided flow chart in the article, 2 in Chinese [22, 29], and 3 in English [8, 14, 26]; potential biases were not described well in the reports; most SRs lack in persuasive outcome measures.

## 4. Discussion

Our overview shows that primary endpoints and secondary endpoints are all used to evaluate the effect of CHM for CHD. Secondary endpoints are most commonly adopted in clinical trials due to their feasibility in small sample size and short-term clinical trials. They may signify future cardiovascular event to some extent and are sure to be valuable as surrogate endpoints. But it is clearly that primary endpoints are more persuasive in RCT of cardiovascular diseases. However, most of the outcome measures in the included SRs are angina pectoris and ECG. Primary endpoints such as mortality and major cardiovascular events are not used widely. Adverse effects, quality of life, and medical economics, which are also important when evaluate a medicine, should be taken as outcome measures too. All of these are the reasons why neither the trials nor the SRs of CHM for CHD could meet a sufficiently high standard to be broadly accepted by the Western medical community.

SRs of CHM with poor methodology and reporting quality have been reported [58]. According to PRISMA statement, we found that most of the included reviews have poor quality. Reviewers were not good at reporting how they avoided bias in selecting primary studies, how they extracted data, and how they evaluated the validity of the primary studies. Also, most of the reviewers chose less persuasive outcome measures, which reduced the persuasion of the interventions. So if reviewers did not master the method of performing SR, they could produce inaccurate or misleading conclusions for current clinical practice and even the future research. Although it appeared that CHM was effective for CHD in clinical use, such as compound salvia pellet, shengmai injection, suxiao jiuxin wan, and gingko, puerarin, most SRs were inconclusive that CHM had a definite effect for CHD owing to the poor evidence.

Before recommending the conclusion, we have to consider the following weaknesses in this overview. Firstly, data were abstracted from SRs instead of the original trials, and most of the included SRs have poor quality. Secondly, most of the RCTs in the SRs included are also of low quality due mainly to unclear randomization and blinding method, incomplete outcome reporting, publication bias, and so forth. Thirdly, we only selected SRs published in Chinese and English. SRs of CHM for CHD published in other language or originated from other countries might be omitted. Fourthly, we did not identify unpublished studies, thus negative trial might not be reported and could induce publication bias.

In conclusion, primary and secondary endpoints were all used to evaluate the effectiveness of CHM for CHD, but primary endpoints were not used widely. Although it appeared that CHM was effective for CHD in terms of some outcome measures, most SRs failed to draw a definite conclusion for the effectiveness of CHM in CHD patients due to the poor evidence. The benefits of CHM for CHD still need to be confirmed in the future with RCTs of more persuasive primary endpoints and high-quality SRs.

## Figures and Tables

**Table 1:** Outcome Measures of CHM for CHD in systematic reviews.

Outcome measures (number of SR)	Condition (number of SR)	CHM	First author	Number of RCTs/total	Conclusion	Risk of publication bias
* Primary Endpoints*						
Mortality (7)	MI (6)	Shenmai injection	Zeng (2010) [7]	13/13	A	H
		Danshen preparations	Wu (2008) [8]	6/6	B	NA
		Shengmai injection	Gao (2008) [9]	4/4	A	NA
		Yiqi huoxue patent medicine	Zhang (2008) [10]	9/28	B	NA
		Herbal injection products	Zhen (2007) [11]	5/15	A	H
		Herbal products	Lin (2006) [12]	4/8	B	L
	Angina pectoris (1)	Tongxinluo capsule	Wu (2006) [13]	1/18	B	H

AMI (6)	MI (2)	Yiqi huoxue patent medicine	Zhang (2008) [10]	1/28	B	NA
		Herbal products	Lin (2006) [12]	2/8	A	L
	Angina pectoris (4)	Compound salvia pellet	Zhang (2008) [14]	1/17	B	H
		Puerarin	Wang (2008) [15]	1/11	B	H
		Tongxinluo capsule	Wu (2006) [13]	3/18	B	NA
		Dengzhanhua injection	Cao (2005) [16]	1/8	A	NA

Restenosis after PCI (1)	CHD (1)	Herbal products	Ren (2008) [17]	17/17	A	H
Recanalization (2)	MI (2)	Yiqi huoxue patent medicine	Zhang (2008) [10]	7/28	B	NA
		Herbal injection products	Zhen (2007) [11]	15/15	A	NA

* Secondary Endpoints (ECG)*						
						
ECG (34)	CHD (4)	Shuyu zaogan tablets	Zhang (2011) [18]	29/32	A	L
		Kudiezi injection	Zuo (2011) [19]	15/16	A	H
	Angina pectoris (30)	Sodium tanshinone IIA Sulfonate	Wang (2011) [20]	17/29	A	H
		Danhong injection	Xu (2011) [21]	19/19	A	H
		Tongxinluo capsule and compound salvia pellet	Jia (2011) [22]	65/58	A	L
		Xuefuzhuyu Decoction	Cui (2011) [23]	8/10	A	H
		Shengmai injection	Zhang (2010) [24]	8/13	A	H
		Safflower Injection	Wu (2010) [25]	2/6	B	NA
		Herbal products	Zhuo (2010) [26]	3/3	A	NA
		Tongxinluo capsule	Hao (2010) [27]	18/20	A	L
		Gingko	Zha (2010) [28]	36/50	A	L
		Gingko	Zhao (2010) [29]	9/23	A	L
		Xuefuzhuyu decoction	Song (2010) [30]	3/3	A	H
		Xinkeshu	Chen (2010) [31]	12/18	A	H
		Yiqihuoxue	Long (2009) [32]	25/30	A	H
		Compound salvia pellet	Zhang (2009) [33]	5/8	A	L
		Compound salvia pellet	Zhang (2008) [14]	10/17	A	H
		Shexiang baoxin wan	Lin (2008) [34]	20/22	A	L
		Puerarin	Wang (2008) [15]	6/11	A	H
		Suxiao jiuxin wan	Wang (2008) [35]	14/14	A	L
		Suxiao jiuxin wan	Duan (2008) [36]	3/15	A	H
		Tong xin luo Capsule	He (2007) [37]	12/17	A	H
		Compound salvia pellet	Jiang (2007) [38]	26/34	A	H
		Danshen preparations	Li (2007) [39]	7/13	A	L
		Compound preparation of salvia miltiorrhiza	Zhang (2007) [40]	30/33	A	H
		Danshen preparations	Li (2007) [41]	20/21	A	H
		Rhodiola L.	Wang (2006) [42]	7/8	A	L
		Tetramethylpyrazine	Zhang (2006) [43]	10/10	A	L
		Tongxinluo capsule	Wu (2006) [13]	10/18	A	NA
		Puerarin injection	Wang (2006) [44]	17/20	A	H
		Compound salvia pellet	Wang (2006) [45]	27/27	A	L
		Dengzhanhua injection	Cao (2005) [16]	8/8	A	NA
		Compound salvia pellet	Wang (2004) [46]	17/17	A	L
		Compound salvia pellet	Zhang (2004) [47]	19/22	A	L

* Secondry Endpoints (Angina Pectoris)*						
		Shuyu zaogan tablets	Zhang (2011) [18]	21/22	A	L
Angina pectoris (30)	CHD (3)	Shengmai injection	Zhang (2010) [24]	10/13	A	H
		Compound salvia pellet	Zhang (2009) [33]	8/8	A	L
		Sodium tanshinone IIA Sulfonate	Wang (2011) [20]	29/29	A	H
		Danhong injection	Xu (2011) [21]	19/19	A	H
		Tongxinluo capsule and compound salvia pellet	Jia (2011) [22]	65/65	A	L
		Kudiezi injection	Zuo (2011) [19]	16/16	A	H
		Shuxuetong	Li (2010) [48]	11/13	A	L
		Herbal products	Zhuo (2010) [26]	3/3	B	NA
		Tongxinluo capsule	Hao (2010) [27]	20/20	A	L
		Xinkeshu	Chen (2010) [31]	16/18	A	H
		Xuefuzhuyu decoction	Song (2010) [30]	3/3	A	H
		Gingko damo injection	Zha (2010) [28]	46/50	A	L
		Ginkgo extract	Zhao (2010) [29]	22/23	A	L
		Suxiao jiuxin wan	Duan (2008) [36]	1/15	A	H
	Angina pectoris (26)	Puerarin	Wang (2008) [15]	10/11	A	H
		Suxiao jiuxin wan	Wang (2008) [35]	14/14	A	L
		Compound salvia pellet	Zhang (2008) [14]	11/17	A	H
		Compound salvia pellet	Jiang (2007) [38]	34/34	A	H
		Compound preparation of salvia miltiorrhiza	Zhang (2007) [40]	32/33	A	H
		Danshen preparations	Li (2007) [41]	21/21	B	H
		Tetramethylpyrazine	Zhang (2006) [43]	8/10	A	L
		Rhodiola L.	Wang (2006) [42]	5/8	A	L
		Tongxinluo capsule	Wu (2006) [13]	5/18	A	NA
		Compound salvia pellet	Wang (2006) [45]	27/27	A	L
		Puerarin injection	Wang (2006) [44]	18/20	A	H
		Dengzhanhua injection	Cao (2005) [16]	8/8	A	NA
		Compound salvia pellet	Wang (2004) [46]	17/17	A	L
		Compound salvia pellet	Zhang (2004) [47]	20/22	A	L
	CHD after PCI (1)	Herbal products	Ren (2008) [17]	15/17	A	H

* Secondry End points (Others)*						
Consumption of nitroglycerine (5)	Angina pectoris (5)	Herbal products	Zhuo (2010) [26]	2/3	A	NA
		Suxiao jiuxin wan	Duan (2008) [36]	1/15	A	H
		Rhodiola L.	Wang (2006) [42]	1/8	A	L
		Puerarin injection	Wang (2006) [44]	6/20	A	H
		Tongxinluo capsule	Wu (2006) [13]	1/18	A	NA

Level of blood lipids (4)	Angina pectoris (3)	Shuxuetong	Li (2010) [48]	4/13	A	H
		Compound salvia pellet	Zhang (2008) [14]	8/22	B	L
		Compound salvia pellet	Zhang (2004) [47]	4/8	A	L
	CHD (1)	Compound salvia pellet	Zhang (2009) [33]	4/17	A	L

Hemorheology (2)	Angina pectoris (1)	Safflower Injection	Wu (2010) [25]	2/6	A	NA
	CHD (1)	Shengmai injection	Zhang (2010) [24]	5/13	A	H

Heart failure (3)	MI (3)	Yiqi huoxue patent medicine	Zhang (2008) [10]	7/28	B	NA
		Danshen preparations	Wu (2008) [8]	1/6	B	NA
		Herbal products	Lin (2006) [12]	3/8	A	L

Arrhythmia (2)	MI (2)	Yiqi huoxue patent medicine	Zhang (2008) [10]	2/28	B	NA
		Herbal products	Lin (2006) [12]	2/8	B	L

UCG (2)	MI (2)	Yiqi huoxue herbal products	Song (2008) [49]	3/3	A	NA
		Herbal products	Lin (2006) [12]	4/8	A	L

Myocardial enzyme (1)	Angina pectoris (1)	Tongxinluo capsule	Wu (2006) [13]	1/18	B	NA

Level of plasma endothelin (2)	Angina pectoris (2)	Puerarin injection	Wang (2006) [44]	2/20	A	H
		Tongxinluo capsule	Wu (2006) [13]	4/18	A	NA

Level of nitric oxide (1)	Angina pectoris (1)	Tongxinluo capsule	Wu (2006) [13]	2/18	A	NA

Heart rate variability (1)	CHD (1)	Compound salvia pellet	Zhang (2009) [33]	3/8	A	L

TCM syndrome (1)	Angina pectoris (1)	Safflower Injection	Wang (2006) [25]	3/8	A	L

Notes: Yiqi huoxue: supplementing qi and activating blood circulation to patients with qi-deficiency and blood-stasis syndrome;

A: CHM may be or appears to be effective; B: The evidence is insufficient, inconclusive;

H: high; L: low; NA: not mentioned.
